# Predicting missing proteomics values using machine learning: Filling the gap using transcriptomics and other biological features

**DOI:** 10.1016/j.csbj.2022.04.017

**Published:** 2022-04-22

**Authors:** Juan Ochoteco Asensio, Marcha Verheijen, Florian Caiment

**Affiliations:** Department of Toxicogenomics, School of Oncology and Developmental Biology (GROW), Maastricht University, Maastricht, The Netherlands

**Keywords:** Proteomics, Transcriptomics, Machine Learning, Rna-sequencing

## Abstract

Proteins are often considered the main biological element in charge of the different functions and structures of a cell. However, proteomics, the global study of all expressed proteins, often performed by mass spectrometry, is limited by its stochastic sampling and can only quantify a limited amount of protein per sample. Transcriptomics, which allows an exhaustive analysis of all expressed transcripts, is often used as a surrogate. However, the transcript level does not present a high level of correlation with the corresponding protein level, notably due to the existence of several post-transcriptional regulatory mechanisms. In this publication, we hypothesize that the missing protein values in proteomics could be predicted using machine learning regression methods, trained with many features extracted from transcriptomics, including known translational regulatory elements such as microRNAs and circular RNAs. After considering different machine learning algorithms applied on two different splitting strategies, we report that random forest can predict proteins in new samples out of transcriptomics data with good accuracy. The proposed pre-processing and model building scripts can be accessed on GitHub: https://github.com/jochotecoa/ml_proteomics.

## Graphical abstract

**Figure:**
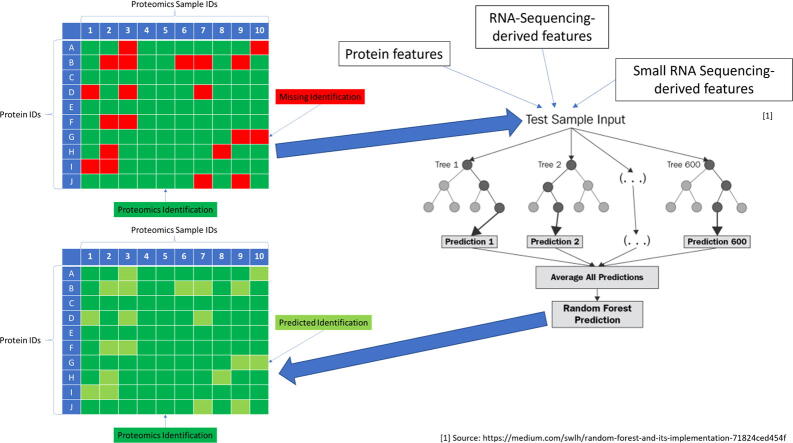

## 1 Introduction

For a cell to react and adapt to any variation of its environment, including for instance the exposure to a foreign compound, a cascade of events leading ultimately to the production of proteins occurs. For that purpose, the cell usually initiates the transcription of its genes (such as transcription factor), and the resulting transcripts containing an open reading frame are translated into proteins. Even though such a schematic view of molecular biology appears straightforward, each of those steps is controlled and affected by a myriad of factors. This complexity led to the development of advanced technologies, named “omics”, allowing to deeply study a particular class of biological entity: transcriptomics (characterization and quantification of transcripts), proteomics (proteins), metabolomics (metabolites), etc.

Among those different classes of molecules, proteins are particularly relevant, as their expression level and activity inform profoundly about how the cell is functioning and reacting to its environment, especially when those changes may pose a risk to the integrity and functionality of the whole system, either due to a disease or an infection. To analyze the expression of proteins in different conditions, proteomics (mass spectrometry or MS) is usually applied. Unfortunately, its sensitivity is limited [1], [2], [3], and thus only a small subset of proteins (with the highest abundance) can be studied at a time. In addition, the stochastic sampling generates missing identifications across samples, particularly for proteins with an abundance close to the detection limit; even though workflows such as DIA (Data-Independent Acquisition)-MS workflow can increase reproducibility. New technologies are not exempt of these limitations: the latest single-cell proteomics strategies (such as SCoPE2^2^) and newest experimental and computational workflows [3] only obtain ∼ 1000 proteins per cell on average (not including their own limitations[4]), even though their dynamic range allows for the quantification of 3000 distinct proteins.

Proteins are mainly translated from messenger RNAs (mRNAs), which are much easier to analyze. Indeed, while having a shorter half-life than proteins, mRNA transcriptomics has become overwhelming sensitive and cost-efficient over the years with the invention of next-generation sequencing. For these reasons, RNA-Sequencing techniques are usually preferred to statistically study cell changes at the molecular level. However, a given mRNA is not an excellent proxy of its corresponding protein expression level, which is reflected in a very low correlation between transcriptomics and proteomics technologies [5], [6], [7], [8], [9]. While the reasons behind this gap can be multiple, the main factors can be categorized into post-transcriptional regulation. By different mechanisms in such regulation, the cell controls the final level of translation of each mRNA into proteins. These factors can be either determined by the molecules themselves (such as the transcript’s or protein’s half-life[10]) or by the interaction with external elements.

MicroRNAs (miRNAs), short non-coding transcripts of around 22 nucleotides of length, play an important role in post-transcriptional regulation. They can act as inhibitors of translation [11], [12] by base-pairing their seed region [13] (nucleotide 2 to 8) to the target mRNA, usually in their 3‘ UTR region. While often considered mild individually, the interaction of multiple miRNAs (either the same miRNA or different miRNAs) on the same 3′UTR target can have a significant effect on protein level expression [14], [15]. Considering the relatively short length of the seed region, miRNAs can target an average of 200 different targets. Even so, miRNAs are not the only transcripts regulating translation.

Another newly discovered category of RNAs, named circular RNAs (circRNAs), are characterized by their circular form, which is generated by the binding of their 5 and 3‘ end during splicing (back-splicing) [16], [17], forming the so-called back-spliced junction. Due to this particular structure, they are not easily degraded due to the absence of transcript extremities, rendering them immune to exonuclease activity [18]. Several functions have been proposed for these circRNAs, including regulating miRNA activities. It has been demonstrated that circRNAs, which can contain repetitions, could present the same target regions present in miRNA targets, and sometimes several times per molecule. This leads to a target competition [19], where circRNAs bind most miRNAs, which gave to circRNAs the function of ‘miRNA sponges’ [20]. The post-transcriptional regulation complexity starts to unfold once one realizes that each transcript can be inhibited by several miRNAs, and at the same time, each of those miRNAs can be “sponged” by one or more circRNAs.

The final expression level of a protein results thus from the integration inside the cell of many factors related to transcripts: the level of expression of mRNAs, the number of possible seeds with miRNAs, the expression level of miRNAs, and the expression level (and “sponging” capacity) of circRNAs able to capture these miRNAs. Many other features could also play a role in this final protein expression level. For instance, the GC content of an mRNA has been observed to interfere with the mRNA half-life [21], and thus the total number of proteins formed from a single mRNA. All these RNA elements or characteristics just mentioned could be identified and quantified by transcriptomics with RNA-Sequencing. Since the protein expression level is the most important factor for biological interpretation, and considering the limited sensitivity and stochastic sampling of proteomics in addition to the very low correlation of the mRNA/protein expression level, we considered the possibility of obtaining predicted protein expression levels from the integration of as many possible features available from several OMICs data. Although we recognized that methods such as match-between-runs (MBR) [22], DART-ID [23], and IceR [24] have already been developed (and their limitations[25]), including a deep learning approach to extrapolate proteomics values from transcriptomics values [26], none utilized a complex multiomics strategy to approach in a novel manner the limitations of proteomics.

The amount and complexity of the data render impossible the task of manually integrating all these parameters. Even when inputting such data digitally, it is not straightforward to visualize which is the optimal manner to predict proteomics values. This problem is characteristic of the current big data era, which in turn, has led to the rise of algorithms that use straightforward optimization strategies to rapidly process thousands or millions of observations. Some of those can be categorized as machine learning (ML), which consists of a set of computer algorithms built to automatically improve their prediction with increasing volumes of data [27]. Specifically, the algorithms focused on predicting are part of the supervised learning algorithms, as they require a training phase in which they are exposed to the value to be predicted (target) in conjunction with other variables associated with it (features). Two major classes of machine learning algorithms exist: when predicting categories or labels (qualitative values), algorithms will perform a classification; while when what is predicted are quantitative values, algorithms will perform a regression. The improvement in the accuracy of these models can be evaluated based on how similar the predictions are to the actual observations. The accuracy is only relevant to evaluate with new data (testing dataset), and not with the data used to train the model (training dataset), in order to avoid the generation of a biased model due to overfitting.

In this manuscript, we hypothesized that using machine learning algorithms would allow us to estimate the expression level of the protein not detected by proteomics out of all available data. For the omics data, we made use of an *in vitro* dataset obtained from primary human hepatocytes microtissues which includes 3 omics datasets obtained from the exact same samples batch: RNA-Seq (*ribo*-depleted libraries), miRNA-Seq (small RNA libraries), and proteomics (mass spectrometry). Both mRNA and circRNAs quantification were extracted from the RNA-Seq data. We thus assessed the accuracy of diverse machine learning predictive models based on different algorithms and data-splitting strategies with the ultimate goal to predict protein expression value from transcriptomics and other mRNA features.

## 2 Methods

### 2.1 Dataset & features

The description of the biological samples used, in addition to the proteomics and transcriptomics protocols followed to obtain protein and RNA expression values, can be found in the Supplementary Methods.

Proteomics expression values were set as the target to be predicted. We set as features protein properties with nominal values extracted from UniProt that might affect their half-life. The features were the following: protein length (Length), mass (Mass), quantity of each amino acid (Aa_X), organism (Organism), location on which the original gene was encoded (Gene.encoded.by), and the database version of the protein sequence (Version..sequence.). From those, we also derived additional features: linear density (mass divided by length) and proportion of each amino acid based on the protein’s length (Aa_X_prop). Finally, we added some irrelevant features (protein sequence version) as negative controls to inform us of the model reliability (based on the importance these features would be given by those models). Concerning protein stability, we included all nine features extracted from the supplementary table: R1-R7, PSI, and SD.

The expression values (in TPM) of protein-related transcripts were added as a feature. Furthermore, we also added diverse transcript properties: strand, transcript length, percentage gene GC content, CDS length, UTR length (or non-CDS length), and proportion of UTR length (UTR length divided by the transcript length). MiRNA expression was also added as a feature, linking it to the transcript targets they could potentially regulate. For this, we used the miRDB’s MiRNA Target Interaction (MTI) score in two features in the ML algorithm: one feature with only miRNAs that presented a high probability of targeting such target (‘stringent‘, score >= 80), and another considering all possible regulations, independently of their score (‘all‘). CircRNA expression as a feature (‘circ’) was linked to the proteomics values based on the miRNA sponging effect of the former. We only utilized the expression of those circRNAs that presented >7 targeting sites with a specific miRNA. We also added the sponging effect of circRNAs as the feature ‘circ_score’.

Transcripts were named based on their Ensembl ID, while proteins were labeled with UniProt IDs. A single UniProt protein could be associated with more than one ENST transcript, potentially with very different features (expression level, transcript length, etc.). Therefore, we needed to summarize the value from all linked transcripts in a single feature. As there was no clear advantage to select a particular summary method over another, we created a feature for each of those different methods: mean, median, minimum, maximum, sum, and standard deviation. This approach was not only applied to features associated with transcripts coupled to proteins (and their log2-transformed values) but also to the ones associated with miRNAs and circRNAs (and their log2-transformed values as well). Indeed, this problem was also applicable to those molecules (to even a greater extent) when linking them to a single proteomics value: each transcript can be inhibited by several miRNAs and each of those miRNAs can be sponged by several circRNAs. We also extended this strategy to those features that presented a multiplicity of values for a single observation, such as the protein stability data. The combination of all discussed variables led to a total of 196 features.

### 2.2 Pre-processing

For both the pre-processing of the data and the construction of the machine learning models, we used the R library ‘caret’ [28].

#### 2.2.1 Creating dummy variables

Since categorical data (such as gender) cannot be inputted directly into a model, they needed to be transformed into dummy variables. Dummy variables are binary features that indicate the presence (1) or absence (0) of a categorical value. In our data, the dummy variables created were related to strand information (positive (+) or negative (−) strand) and protein version sequence (presence or absence of versions 1 to 7).

#### 2.2.2 Identifying (Near) Zero-Variance and correlated predictors

To identify variables with no variance (Zero-Variance or ZV) or insignificant variance (Near Zero-Variance), we used the function ‘nzv’ (frequency ratio > 95/5, percentage unique < 10%) described in ‘caret’ [29]. We then discarded those predictors from the dataset. To identify correlated variables (correlation > 0.75), we used the function ‘findCorrelation’ also from the ‘caret’ package. We discarded the identified correlated predictors from the dataset. The correlation plot was designed using the ‘corrplot’ package.

#### 2.2.3 Centering and scaling

Centering refers to the data transformation where the means of all features are set to a specific value (i.e., 0) while scaling refers to the transformation where the standard deviation is also set to a constant value (i.e., 1). These data transformations avoid a feature importance bias due to value size or scale. No imputation was performed, but instead, all observations with any missing value were removed from the dataset.

#### 2.2.4 Data splitting and algorithms used

The data split between the training dataset (80% of the whole dataset) and the testing dataset (20% of the whole dataset) was performed based on 2 different strategies: sample names and protein names. For each algorithm used, we performed recursive feature elimination using the ‘rfe’ function with (10-fold) cross-validation (CV) resampling and the training dataset. After recursive feature elimination, the model with the optimal subset size of variables for each algorithm was selected to predict the testing dataset. As validation, we also used ‘rfe’ (10-fold cross-validation) for the whole dataset. To split the dataset accordingly, we first generated the 10 folds using the ‘groupKFold’ function based on the indicated categories (samples and proteins). These folds were used as input in the ‘folds’ parameter in the ‘rfeControl’ function.

The algorithms tested were: Boosted Tree (‘bstTree’), Random Forest (‘rf’), Bagged Model (‘bag’), Boosted Tree (‘blackboost’), Lasso and Elastic-Net Regularized Generalized Linear Model (‘glmnet’), k-Nearest Neighbors (‘kknn’), Cubist (‘cubist’), and Linear Regression (‘lm’). All algorithms were used via ‘caret’, and thus, the default parameters used by ‘caret’ were utilized.

#### 2.2.5 Performance based on GO terms

We selected the cardiac dataset, and subselected one sample as testing dataset, while the model training was proceeded with the rest of samples using the 10 features shown in the results. After the training, we predicted the testing dataset with the resulting random forest model, and combined the predictions with the testing observations. We then extracted the GO terms associated for each protein in the testing dataset, which we also combined with the observations and predictions. We discarded GO terms that were categorized in less than 10 proteins. We evaluated the R^2^ metrics for each of the groups of proteins associated to each GO term. We ranked the GO term groups from best to worst performing based on R^2^. All the code used can be located in the following script on GitHub: ‘script/go_terms_analysis/rsquared_on_different_go_terms.R’.

#### 2.2.6 Imputation: A potential use of the random forest model

We also selected the cardiac dataset, but in this case including all proteomics missing values. We subselected all Untreated (UNTR) samples. The training dataset only contained observations with quantified proteomics values, and the 10 features mentioned in the results. We used the random forest algorithm for the training of the model. We then predicted the missing proteomics values using the newly trained model. We combined the results with the observed data, and sampled proteins with different proportions of missing data. All code run can be found in the following script on GitHub: ‘script/imputation/imputing cardiac values.R’.

## 3 Results

To assess the ability of the regression ML algorithm to estimate the level of proteins, we produced a dataset that presented the added value of having transcriptomics (both *ribo*-depleted and small RNA libraries) and proteomics (LC/MS), all generated from the exact same sample batches to maximize the interpretability of the interactions. This dataset was composed of a total of 115 *in vitro* samples (61 cardiac and 54 hepatic). The processing of all these samples (Methods) characterized an amount of expressed biological entities summarized in Table 1. The total number of expressed biological entities was 48 266 and 48 715 for the hepatic and cardiac tissues respectively.

**Table 1:** Summary table of all quantified biological entities. Total refers to all possible entities to be identified. Expressed (N) refers to the number of entities that were quantified in at least 1 sample. Constitutive (N) refers to the number of entities that were quantified in all samples. Expressed (%) and Constitutive (%) refer to the percentage of (constitutively) expressed entities based on the total number of entities. Constitutive (% Expressed) refers to the percentage of constitutively expressed entities based on the number of expressed entities.

	Tissue	Total	Expressed (N)	Constitutive (N)	Expressed (%)	Constitutive (%)	Constitutive (% Expressed)
Proteomics	Hepatic	1806	1806	283	100.00%	15.67%	15.67%
Proteomics	Cardiac	2217	2217	247	100.00%	11.14%	11.14%
Linear transcripts	Hepatic	211,939	135,655	894	64.01%	0.42%	0.66%
Linear transcripts	Cardiac	211,939	136,860	933	64.58%	0.44%	0.68%
MicroRNAs	Hepatic	2744	1561	280	56.89%	10.20%	17.94%
MicroRNAs	Cardiac	2744	1510	250	55.03%	9.11%	16.56%
Circularized transcripts	Hepatic	140,317	95,106	151	67.78%	0.11%	0.16%
Circularized transcripts	Cardiac	140,317	100,416	156	71.56%	0.11%	0.16%

To assess the possibility to predict protein expression levels for all genes using ML algorithm, we needed to assemble a list of features, either parametric or categorical. From all the Table 1 data, we extracted 12 features focused on the expression level of linear transcripts, 24 features on miRNA expression, and 12 features on circular RNA expression. We added 36 features on transcript characteristics (strand, transcript length, etc.), 48 features on protein characteristics (Protein Mass, Protein Length, etc.), 12 features on MTI (miRNA target interaction) scores, a feature on RNA-Sequencing depth, 6 features on circular scores (number of miRNA binding site per circular RNA), 12 features on circular RNA expression, and 45 features on protein stability. This led to a total of 196 features on the raw dataset. Even so, some of those features might be deemed irrelevant due to their multiplicity and inherent structure. Those features might affect machine learning processes, depending on the algorithms’ inherent functionality, by decreasing their accuracy [30]. To avoid their inclusion, we applied several pre-processing filters that removed non-informative features, which are described below.

### 3.1 Zero- and near zero-variance variables

Some predictors can have a unique value for all observations (Species: Human), which can make models unstable or decrease their fitness. Those features can be named as Zero-Variance (ZV) variables, and they are generally removed. Similarly, Near Zero-Variance (NZV) variables refer to features that present a value in an overwhelming majority of observations (i.e., genes coded in the nucleic genome vs genes coded in the mitochondrial DNA (Table 2)). These features are generally not helpful in a cost/benefit ratio, as the underrepresented values might have an artificially bigger impact, and these values may not even appear in the subpopulations generated by sub-sampling strategies, generating a ZV variable (Table 3).

**Table 2:** Examples of Zero- and Near Zero-Variance variables. The ‘Organism’ variable contains a single unique value (‘Human’), thus this value has no predicting value. The ‘Gene encoded by’ variable contains 2 possible values, of which ‘Nucleus’ represents>99% of all observations. Even though this variable does indeed have more than a single value, the frequency of its values renders it non-informative.

Protein ID	Organism	Gene encoded by
A – Sample 1	Human	Nucleus
B – Sample 1	Human	Nucleus
C – Sample 1	Human	Nucleus
D – Sample 2	Human	Nucleus
E – Sample 2	Human	Nucleus
F – Sample 2	Human	Nucleus
G – Sample 3	Human	Mitochondrion

**Table 3:** Examples of ZV and NZV features with their respective frequency ratios and unique percentages. The metrics for all NZ features were identical, as they only reported a single value (Inf: Infinite). For NZV values, they all presented a frequency ratio above 19 (95/5) and a percentage unique below 10.

Feature name	freqRatio	percentUnique	zeroVar	nzv
circ_min	Inf	0.004	TRUE	TRUE
circ_min_log2	Inf	0.004	TRUE	TRUE
Organism	Inf	0.004	TRUE	TRUE
strand_sd	839.433	0.008	FALSE	TRUE
transcript_length_sd	140.014	3.494	FALSE	TRUE
percentage_gene_gc_content_sd	515.167	0.107	FALSE	TRUE
cds_length_sd	64.757	2.098	FALSE	TRUE
noncds_length_sd	112.344	3.427	FALSE	TRUE
proportion_noncds_length_sd	336.033	3.693	FALSE	TRUE
Gene.encoded.by	1198.048	0.008	FALSE	TRUE

Due to both ZV and NZV filters, 44 features were removed from the dataset. Only a few were labeled as ZV, examples of which were ‘circ_min’ (minimum circular expression) and its log2 transformed version ‘circ_min_log2′. Some categories of variables were frequently labeled as NZV: almost all features related to miRNA scores; all maximum, median, and minimum miRNA expressions (non-transformed, log2-transformed, stringent, and all scores); some related to circular scores and some related to circular expression (Supplementary Table 1).

### 3.2 Identification of correlated variables

Having correlated predictors is generally uninformative and sometimes detrimental to build models. For this reason, we removed features that presented a correlation above 0.75. For each pair of correlated features, the feature labeled as ‘highly correlated’ was the one that presented a higher correlation with the rest of the variables. Having the target inside the dataset would imply that the features that showed a higher correlation with the target would get removed. To avoid this, we removed the target from the dataset before filtering the highly correlated variables. In total, 93 features were removed due to high correlation (Supplementary Table 2). As expected, the abundances of most amino acids were highly correlated to each other, and to the protein mass and length. The same results were not true for the proportion of each amino acid, as they more accurately represent their presence independently of the protein’s size. More surprisingly, among all non-filtered features, we observed all possible grouping systems (minimum, mean, median, maximum, standard deviation, and sum of the values they represented), with no clear predominance for any of them, and thus none appeared to present a tendency to be the most informative (i.e., the one with the lowest overall correlation with all features).

### 3.3 Splitting strategies

In both hepatic and cardiac datasets, the observations were part of two distinct groups: proteins and samples. Having a random split of our data to form both training and testing datasets would not have enabled us to elucidate the actual accuracy of the model. In a random splitting strategy, the training dataset was highly probable to include most proteins and samples in their observations, rendering the data split futile. Instead, we split (and trained) our models separately in two manners: splitting by sample and splitting by protein (Fig. 2). This strategy was applied in the hepatic dataset for both training and testing, and in the cardiac dataset for validation.

**Fig. 1:**
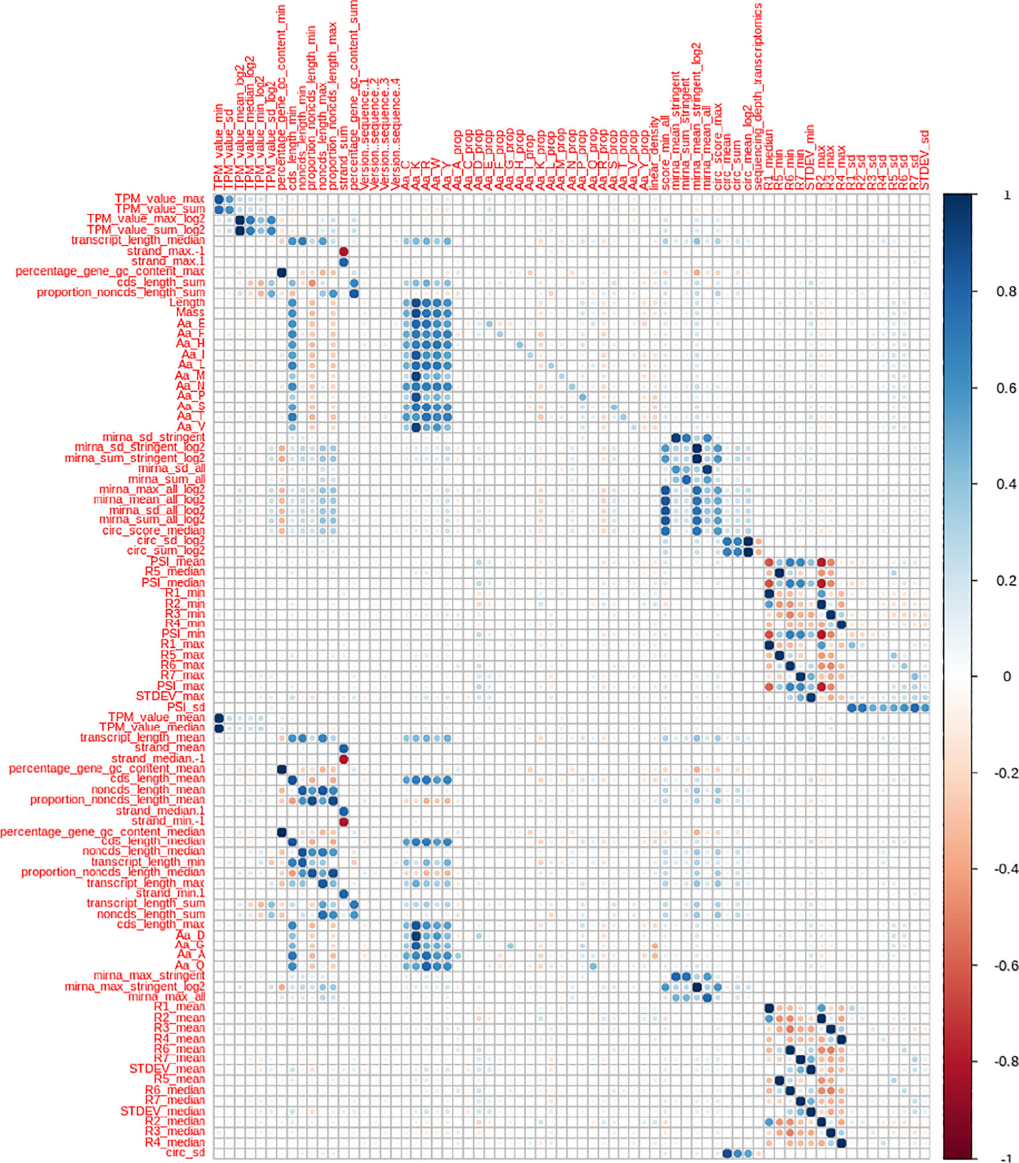
Correlation plot between kept features (horizontal axis) and filtered features (vertical axis). The scale unit on the right side of the figure indicates the correlation values between the features shown based on a range of colors: from dark red (extreme negative correlation) to dark blue (extreme positive correlation), where lighter colors represent a lower absolute correlation value. (For interpretation of the references to colour in this figure legend, the reader is referred to the web version of this article.)

**Fig. 2:**
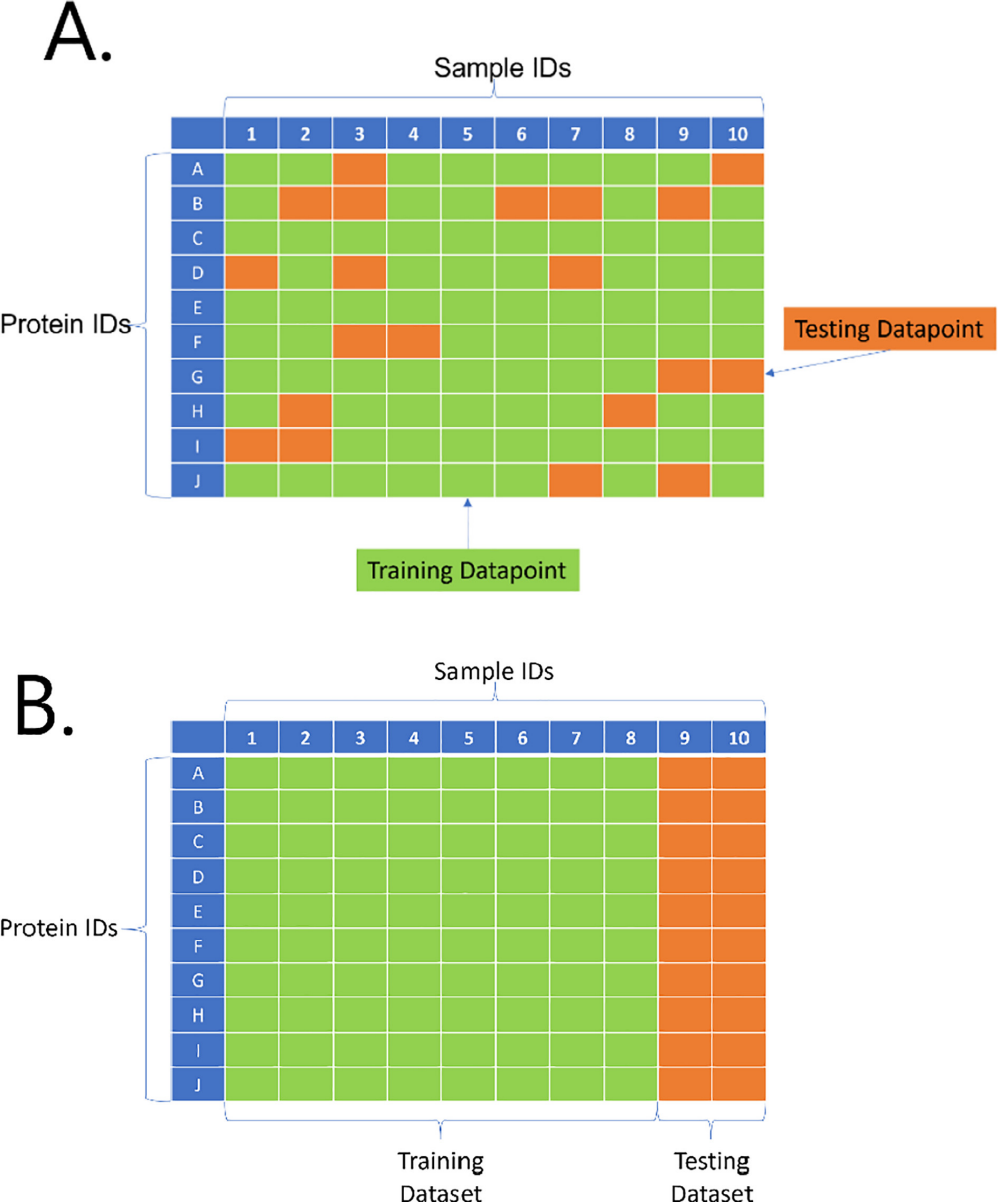

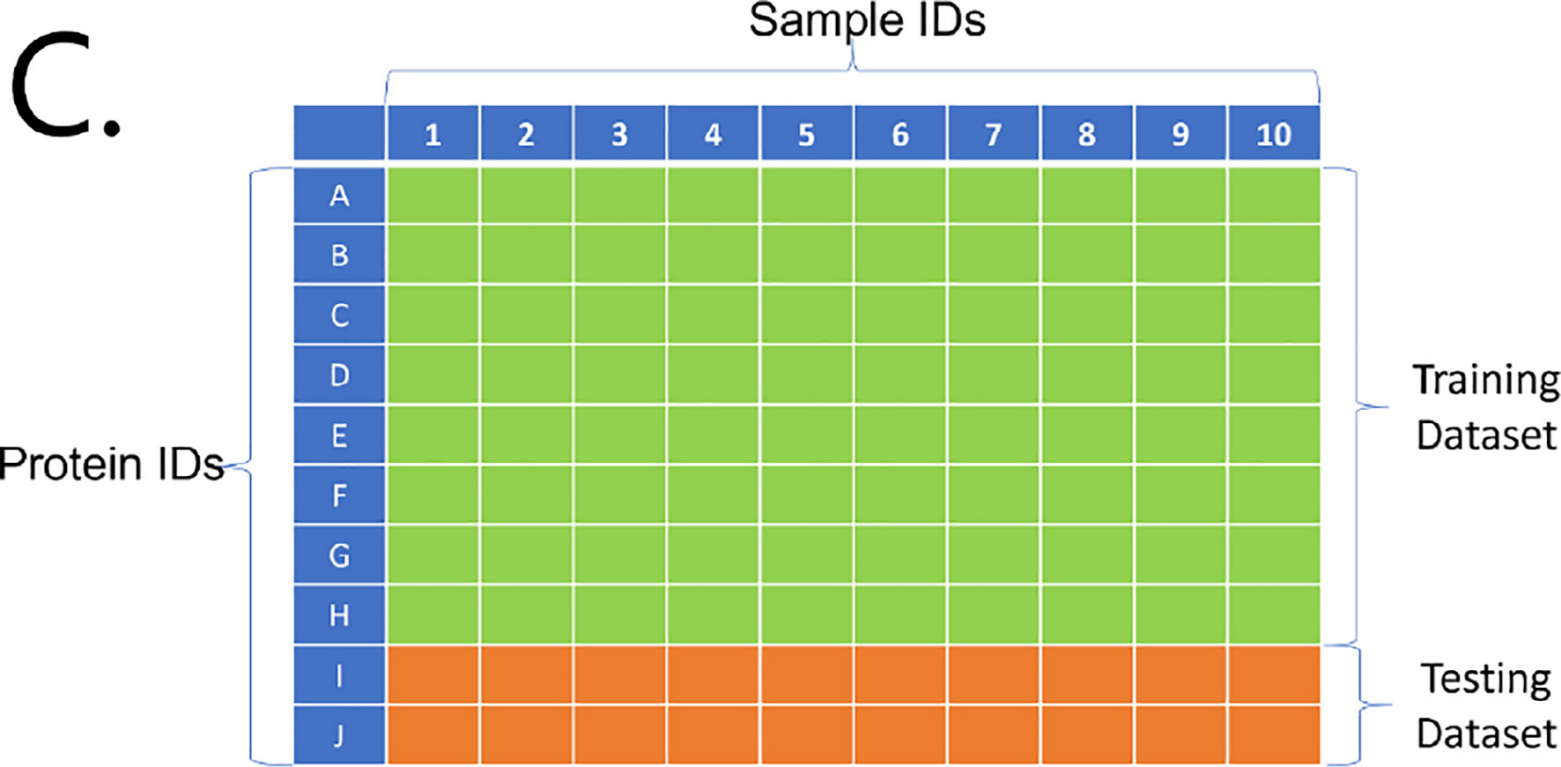
Splitting strategies. For all splitting strategies, 80% of the data is used to train the models (training dataset), while the other 20% is used for testing the trained models (testing dataset). A. Random splitting strategy, where the algorithm is trained and tested with observations from all proteins and samples. B. Sample-splitting strategy: the trained models are tested with 20% of the samples. C. Protein-splitting strategy: the trained models are tested with 20% of the proteins.

### 3.4 Model training and testing using hepatic sample-split data

**Fig. 3:**
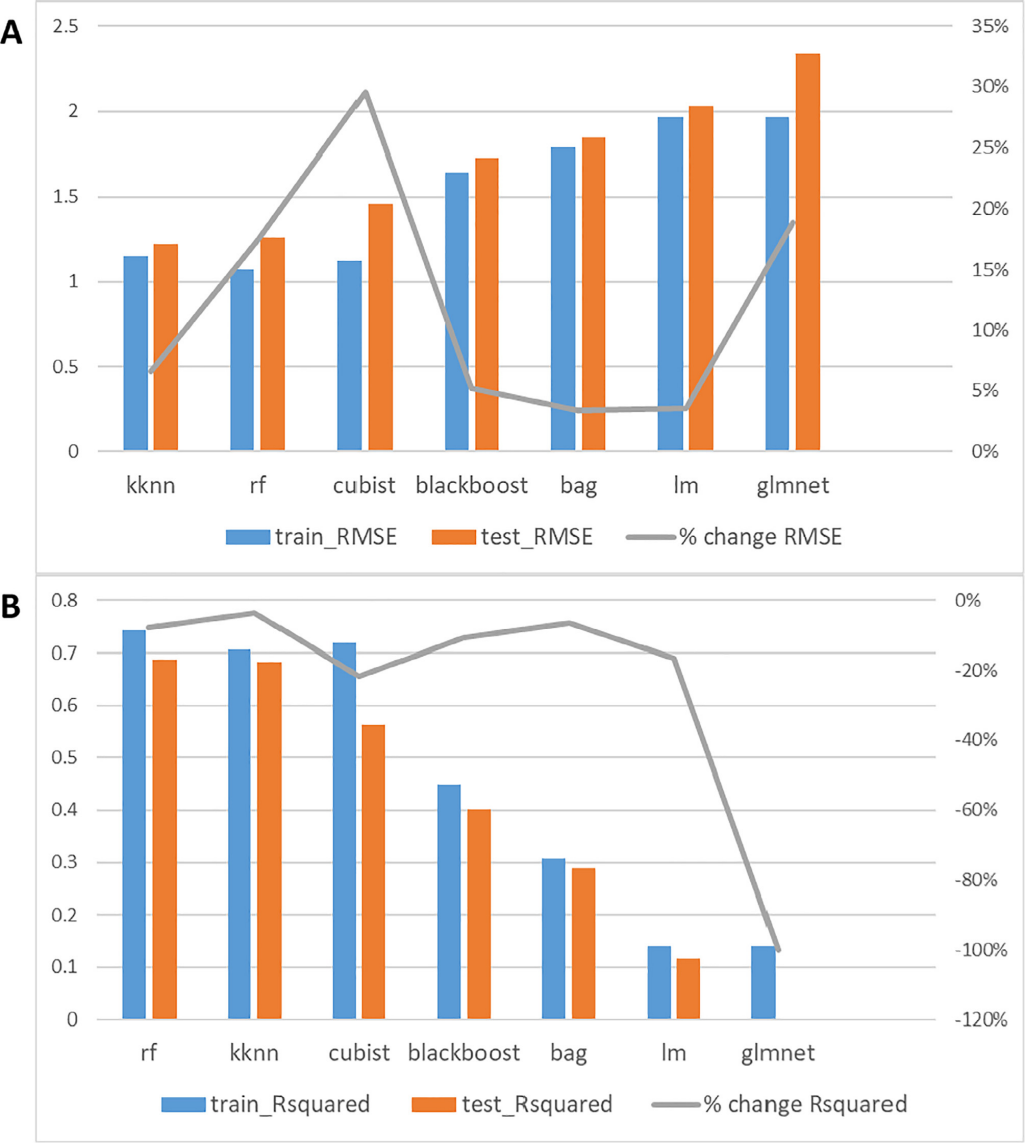
Accuracy results when splitting by sample. A: The blue bar refers to the RMSE value (left vertical axis) after training the model with 80% of the samples, and the orange bar refers to the RMSE value after testing the model with the other 20% of the samples. The gray line refers to the percentual change of RMSE (right vertical axis) between training and testing. B: The blue bar refers to the R^2^ value (left vertical axis) after training the model with 80% of the samples, and the orange bar refers to the R^2^ value after testing the model with the other 20% of the samples. The gray line refers to the percentual change of R^2^ (right vertical axis) between training and testing. (For interpretation of the references to colour in this figure legend, the reader is referred to the web version of this article.)

When splitting by sample, 80% of hepatic samples were used as the training dataset, while the other 20% was used as the training dataset. For every algorithm, the training dataset was inputted through RFE (cross-validated with 10-fold). Out of all models trained with different subsets of features, the one with the best accuracy was used for the testing step (Fig. 3). In terms of root-mean-square error (RMSE, Fig. 3A), both k-Nearest Neighbors (‘kknn’) and Random Forest (‘rf’) showed the highest accuracies (∼1.25), the latter having a bigger deviation between training and testing RMSE values. To evaluate these results in a more standard and informative manner, we also analyzed the R squared metrics (Fig. 3B). In this figure, we observed that rf and kknn also showed the best performance (R^2^ close to 0.7), showing rf better performance in this case.

**Fig. 4:**
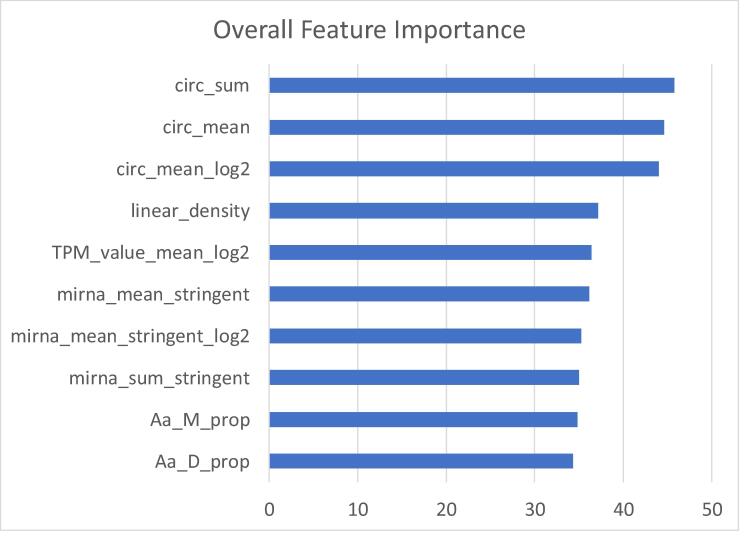
Top 10 features based on Overall importance by RFE when using the rf algorithm. These values represent how important (on average) each feature is to the model, and thus which are the main features used by the model to predict new proteomics values.

After validating the aforementioned results by using RFE (10-fold cross-validation) for the whole dataset (Supplementary Figs. 1 and 2), we selected random forest (‘rf’) as the best performing model when splitting by sample. The optimal subset size of features was 51 features, but after close examination of the RFE results (Supplementary Fig. 3), we determined that subset sizes above 10 features had a minimal impact on RMSE. The 10 features were selected based on the ranking of feature importance reported by the RFE analysis (Fig. 4).

### 3.5 Model training and testing using hepatic protein-split data

**Fig. 5:**
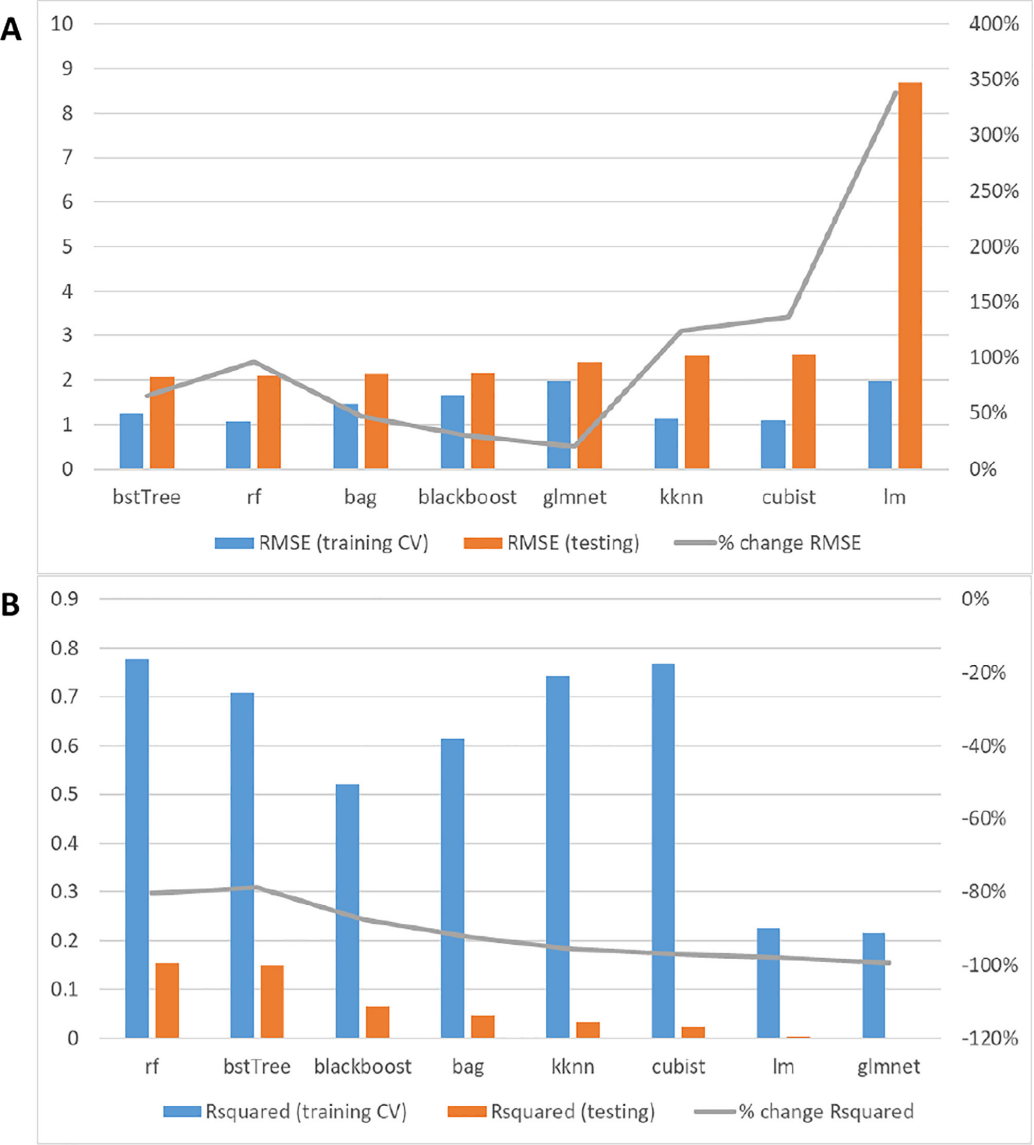
Accuracy results when splitting by protein. A: The blue bar refers to the RMSE value (left vertical axis) after training the model with 80% of the proteins, and the orange bar refers to the RMSE value after testing the model with the other 20% of the proteins. The gray line refers to the percentual change of RMSE (right vertical axis) between training and testing. B: The blue bar refers to the R^2^ value (left vertical axis) after training the model with 80% of the proteins, and the orange bar refers to the R^2^ value after testing the model with the other 20% of the proteins. The gray line refers to the percentual change of R^2^ (right vertical axis) between training and testing. (For interpretation of the references to colour in this figure legend, the reader is referred to the web version of this article.)

Similar to the splitting by sample strategy, a fifth of all proteins were split to be used as the testing dataset, while the other 4 fifths were used as the training dataset. RFE (10-fold CV) was also performed with similar optimal results as in the training dataset of the sample-splitting strategy (Fig. 5). In this case, the best RMSEs in the testing dataset include ‘bstTree’ and ‘rf’ (≈2), which almost doubled the error shown when splitting by sample (Fig. 5A). To understand how relevant this error increase was, we also evaluated the R-squared values of those values (Fig. 5B). We observed that a systematic gap existed between the training and testing steps, leading to minimal R-squared values (R^2^ = 0.15 for rf).

For all the results shown above (Fig. 3, Fig. 4), we also validated the results using RFE with the whole dataset (no training–testing split), where the folds or splits in the cross-validation step (10-fold) contained exclusively a set of proteins (Supplementary Figs. 4 and 5).

### 3.6 Random forest model validation with a cardiac sample-split data

Random forest being the best performing model, we decided to validate its accuracy to predict new samples using a cardiac dataset, which was built in the same manner as the hepatic one. The validation included using the same algorithm (rf) with the same top 10 features (Fig. 4), and training and validating it with the cardiac data (27602 observations). The resampling was performed via Cross-Validation (10-fold). Using the cardiac data and the specified model, we validated that the accuracy remained robust across different cell types (RMSE = 1.04, R^2^ = 0.75; Supplementary Fig. 6).

**Fig. 6:**
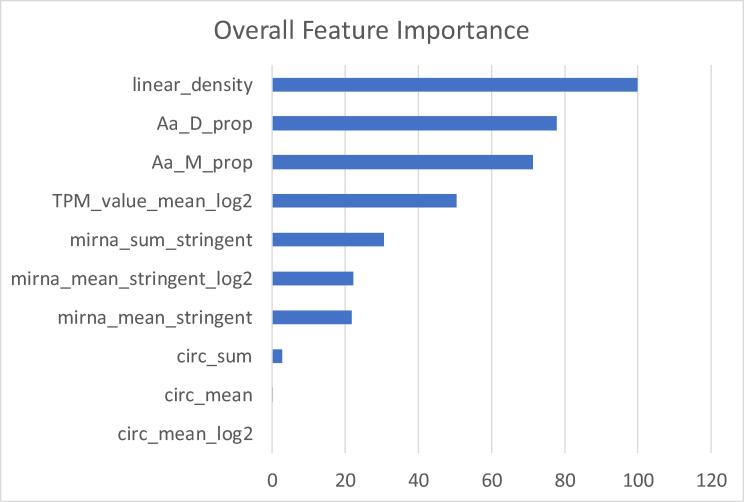
Feature Overall Importance for the rf algorithm when trained in Cardiac data. These values represent how important (on average) each feature is to the model, and thus which are the main features used by the model to predict new proteomics values.

The only remarkable difference was the feature importance ranking given by the RFE in the hepatic data (Fig. 4) compared to the feature importance ranking given by the model itself with the cardiac data (Fig. 6). In the latter, linear_density is given the utmost importance, and the importance of the three RNA subtypes relate to how close they are to the protein level: mRNA level, followed by miRNA levels, and finally circRNA levels.

Therefore, the RMSE and R-squared metrics for both cardiac and hepatic models showed that building a random forest model using the aforementioned features allowed to predict with high accuracy full proteomics’ samples. Comparing the testing results between sample- and protein-splitting, we observed that the high accuracy was especially due to the prediction of proteins that have already been trained on. Observing the feature importance ranking (Fig. 6), we could observe that different biological entities presented a different relevance to the model’s accuracy, thus missing some variables will have a minimal effect on the decided outcome.

### 3.7 Performance based on GO terms

**Fig. 7:**
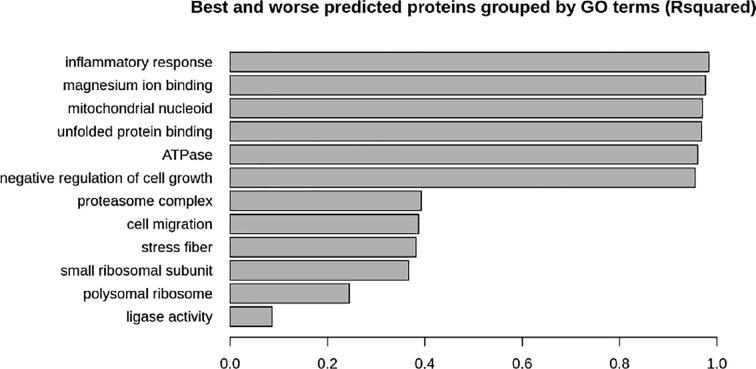
R^2^ results categorized in GO terms. The X axis represents the R^2^ values, while the bar labels represent the GO terms. Only the 6 best and 6 worst GO terms are depicted.

Even though we obtained good substantial results for the prediction of proteomics values at a sample level, these results were an overall representation of all proteomics values, and thus did not inform which protein groups would be better or worse predicted by our model. For this reason, we decided to stratify the predictions based on GO terms, and then evaluate their R^2^ metrics when compared to their counterpart observations. The overall metric for the testing data/sample in this experiment was R^2^ ∼ 0.82. What we observed (Fig. 7) is that there were considerable differences in R^2^ depending on the GO term the proteins were associated to. While the 6 best-performing GO terms (inflammatory response, magnesium ion binding, mitochondrial nucleoid, unfolded protein binding, ATPase, and negative regulation of cell growth) had near perfect results (R^2^ > 0.9), the worst performing ones (ligase activity, polysomal ribosome, small ribosomal unit, stress fiber, cell migration, and proteasome complex) showed metrics half the performance shown in the overall results (R^2^ ≤ 0.4).

### 3.8 Imputation: A potential use of the random forest model

As the model showed a promising accuracy for predicting whole replicate samples, we hypothesized that the model could also be used for imputation of missing values for proteins that were at least present in one of the samples of the training data. To showcase a possible example, we trained a random forest model with all the Untreated samples (UNTR) and the corresponding 10 features. The example (Table 4) showed that the proteomics values imputed fitted the range of quantification observed in the quantified values of the same protein, while differing from each other from sample to sample. We also observed that in these samples, values tend to be missing simoultaneously for samples taken at the same time.

**Table 4:** Imputation of Proteomics Cardiac samples. Every row is identified with a UniProt ID, and represents a protein quantified in at least one of the untreated samples of the cardiac dataset. Each column represents each Untreated (UNTR) sample from the cardiac dataset. On the column names, the first number represents the hour at which the sample was taken (2 h, 8 h, etc.), while the second identifies the replicate number (002_1 was the first replicate sample taken after 2 h). The proteins (rows) are sorted by proportion of missing data in a increasing order. Values with a dark green background were quantified by proteomics. Values with a light green background were imputed/predicted by the random forest model.

## 4 Discussion

We wanted to build a machine learning model that tightened the gap between transcriptomics and proteomics, using the former as a predictor of the latter. The results indicate that a random forest model, by using only 10 features, can predict with good accuracy (R^2^ = 0.74) proteomics values from samples in similar circumstances to the ones where it has been trained on. However, predicting protein expression by training the model on other proteins was highly inefficient (R^2^ = 0.15).

Interestingly, 7 out of the 10 features used by the model were related to RNA expression (Fisher's Exact Test for Count Data, p-value = 0.0027). Out of these 7, the most important (as expected) was mRNA expression, which is directly linked to translation, and thus, to protein expression. Followed in feature importance came 3 features related to miRNA expression, which is known to inhibit translation to a vast number of coding transcripts. The least important features related to the 3 RNA subtypes referred to circular RNA expression. Circular RNAs have been hypothesized to work as miRNA sponges, and so even though they are involved in post-transcriptional regulation, they have a more indirect effect. It is postulated that most circular RNAs are by-products of faulty splicing [31], and thus their regulation might just be mainly due to the regulation of their host gene. Even so, their consistent expression would still allow them to have an impact on post-transcriptional regulation.

Linear density (mass of a protein divided by its length) and the proportions of both Aspartic Acid and Methionine were the most important features for the final random forest model. One hypothesis to explain such model behavior was that these three features (and especially linear density) helped to categorize observations protein-wise: an observation with similar values across the three top features could be likely categorized as a similar protein, and thus, also presenting a close expression value. This already made the model highly accurate when trained and tested with similar samples. The other features (related to the current transcript expression level) might have helped to succinctly tune the protein expression already observed in similar proteins during the training step. Another hypothesis, only relevant to linear density, was linked to the proteomics technology itself: linear density was directly linked to protein mass, which is used (along with charge) to identify and quantify protein in mass spectrometry; hence, its relevance as a feature. In addition, having linear density as one of the main features underlines the importance of the training data for our model. A random forest model can only predict values learned beforehand, thus we hypothesize that linear density helps the model to find the most similar protein when predicting. Thus, the use of this model should be to predict proteins that are already quantified in some of the samples, limiting the effect of potential false positives, and therefore also limiting the potential false biological significances created by false positives due to differences that only occur at the transcriptional level.

The observed divergence between the feature importance ranking in RFE and the validation model may be due to how RFE evaluates features while using cross-validation. At the beginning of the process, RFE built 10 different training–testing combinations (based on the 10 folds), and, based on the initial ranking of all the features in each of those combinations, features were removed from least to most important. Each feature was ranked based on the average of all the rankings performed during the feature elimination. In the validation model, instead, the feature importance ranking represented the concrete importance of each variable for that specific model and algorithm.

Considering the relatively high accuracy of the random forest model to impute protein expression from a reduced subset of features, we see an application of this proposed strategy to contribute to compensating for the lack of depth of proteomics. Indeed, since proteomics only allows the analysis of a subset of proteins per sample, with usually only a partial overlap between samples (even at the replicate level), our model would be able to predict and fill those values, increasing the strength of the statistical analysis of such proteins across treatments.

However, as shown in the GO-term-performance results, the metrics are not uniform for all categories of proteins, and this should be taken into consideration when performing analysis with a specific focus on a certain protein category. This difference may be the result of three different causes: 1/ the correlation of protein abundance with their coding RNA levels may differ across GO categories, 2/ as different GO categories contained an unequal number of proteins, the size of a GO category was inversely proportional to the R^2^ metric (a smaller random set of values has a higher chance of obtaining a high R^2^, and vice versa), 3/ GO categories with stable protein abundances (and mRNA levels) performed better than otherwise.

An important detail to consider is that drastically different data is generated when utilizing different methods to quantify proteomics intensities: from values that correlate with absolute abundance based on the MS signal of histones (also referred to as the “proteomic ruler” approach [32]), going through intensities inferred based on the ratio of detected peptides (pertaining to each protein) between samples (MaxLFQ [33]), to isobaric proteomics data (TMT/iTRAQ); wherein changes in the peptide intensity from one sample has a ripple effect on the intensities from all the co-isolated samples [34]. In our study, the Hi3 label free method [35] was used to quantify protein intensities, hence values from absolute abundance methodologies are expected to perform similarly. Despite that, isobaric proteomics methods should not be entirely dismissed, as the range of values predicted by a random forest model is highly dependent on the range of the data the model is trained on. The compositional nature of isobaric proteomics experiments results in signals that are highly batch-dependent. Our predictions would not take the batch structure into account, and as a result, a correction would be required. Thus, the inability of random forest models to extrapolate does make them an appealing option for compositional data, but simoultaneously may be a limiting factor for absolute intensity values.

Based on the inefficient accuracy for all models tested in the protein-splitting strategy, we hypothesize that even though we tried to include as much information related to protein expression as possible (transcript expression, transcript properties, protein characteristics, and stability), predicting protein expression anew (without ever training the model with that protein’s data) may have required of an even more complete (i.e. RNA binding proteins, long non-coding RNAs, transcript half-life, etc.) or different set of features. For example, a study by Barzine et al. [26] showed improved results (R^2^ = 0.51) extrapolating proteomics values while only using gene expression data, GO terms, and UniProt keywords. Future research should focus on either including the last two features as features to the dataset, or improving their deep learning model by including our (or other) post-transcriptional features.

In conclusion, after developing different machine learning models to predict proteomics values out of transcriptomics ones, we have achieved to build a random forest model that can predict with significant accuracy the protein expression of a new sample. Building a random forest model with the selected features can thus be used to predict the missing data inherent in proteomics studies, independently of the cell’s nature. The code used for the pre-processing of data and the model building process is available on Github (https://github.com/jochotecoa/ml_proteomics) [36].

Data availability.

Data has been submitted to the BioStudies repository (https://www.ebi.ac.uk/biostudies/) and is available under the following accession numbers:

Hepatic data: S-HECA33, S-HECA34, S-HECA47, S-HECA158, S-HECA457, S-HECA460, S-HECA463.

Cardiac data: S-HECA1, S-HECA9, S-HECA18, S-HECA139, S-HECA447, S-HECA449, S-HECA453.

## Declaration of Competing Interest

The authors declare that they have no known competing financial interests or personal relationships that could have appeared to influence the work reported in this paper.
