# Supplementary material for: Predicting missing proteomics values using machine learning: Filling the gap using transcriptomics and other biological features
Source: Comput Struct Biotechnol J. 2022 Apr 22;20:2057–69. doi: 10.1016/j.csbj.2022.04.017 (PMC9077535; doi:10.1016/j.csbj.2022.04.017)
Supplement: Supplementary data 1 [file mmc1.docx]

SUPPLEMENTARY METHODS

# Biological Samples

The omics data used for building the models were obtained from 2 different human 3D microtissues from InSphero. The first dataset consists of primary hepatocytes (61 samples), while the data from the second one was obtained from iPSC (Induced pluripotent stem cell) cardiac cells (54 samples). All samples were either simply cultivated in medium (untreated) or exposed to the solvent DMSO. Enough microtissues were produced for each batch to generate the data for 3 different omics technologies: RNA-Seq, miRNA-Seq, and MS proteomics. Any sample that contained a low coverage in any of the omics technologies was removed from the analysis entirely. A low coverage sample was defined as a sample whose sequencing depth was 4 times lower than the average sequencing depth of all samples.

# Proteomics

Protein samples were subject to isolation and dilution to a concentration below 0.2M. Afterward, they were digested by trypsin, followed by a cleaning-up using Sep-Pak tC18 cartridges (Waters) based on the manufacturer’s instructions. The peptides were dried in a vacuum centrifuge and measured on an Orbitrap Fusion mass spectrometer (Thermo Fisher Scientific), coupled to a NanoLC-2D HPLC system (Eksigent). The raw MS data were processed using Genedata Expressionist software (v.11.0). LC-MS peaks were noise-reduced and normalized, followed by the collection of their properties (m/z and RT boundaries, m/z and RT center values, intensity). Mascot 2.6 was used for the annotation of the individual MS/MS spectra. The peak clusters grouping was performed via protein interference (using peptide and protein annotations), and protein intensities were obtained with the Hi3 method. Proteomics data, which was the target to predict, was normalized using the median of medians approach^1^. Each observation referred to the expression of a specific protein in a specific sample. Proteomics values, due to their normalization strategy, are log2-transformed versions of the original values, which transforms the distribution of those values from a negative binomial distribution into a Gaussian one.

We also utilized protein stability data from Hsueh-Chi Sherry Yen et al.^2^, specifically the supplementary Table S2: the complete list of protein stability profiles in 293T cells. In this study, the authors fractioned the cell library into seven subpopulations based on their fluorescence, representing the relative stability of a specific protein per cell: R1 (unstable) to R7 (very stable). Not all cells with the same fluorescent protein presented the same fluorescence, thus the values found below each fraction (R1 to R7) represented the proportion of cells with such fluorescent protein in that fraction (the sum of all proportions for each protein equaling to 1). The table also included the “protein stability index” (PSI), which ranged from 1 to 7, with a higher PSI value corresponding to a higher relative protein stability; and the standard deviation (SD) to evaluate how much the stabilities of a protein varied across all cells where that fluorescent protein was expressed.

# Transcriptomics

Total RNA was isolated from the microtissues with the Qiagen AllPrep Universal Kit (Cat #80224). The samples were ribo-depleted by using the Illumina RiboZero Gold kit (Cat #MRZG12324), and their library preparation was performed using the Lexogen SENSE total RNA kit (Cat #009.96). The paired-end sequencing of all libraries was generated on an Illumina HiSeq 2000 at 100 bp, with an average coverage between 20 and 30 million reads. We used trimmomatic^3^ for trimming the RNA-Seq FASTQ files using the following parameters: paired-end, ILLUMINACLIP: TruSeq3-PE.fa:2:30:10, LEADING:3, TRAILING:3, SLIDINGWINDOW:4:15, MINLEN:36, HEADCROP:12. Later we quantified transcript expression using Salmon^4^. Salmon’s transcriptome file also contained the circBase IDs and sequences of human circular RNAs. We extracted the TPM (Transcripts Per Million) values from each of the samples as the normalized transcript observation.

For the miRNA-Seq, an aliquot from the same total RNA isolated for the ribo-depleted libraries was size selected and ligated using the TruSeq Small RNA Library Prep Kit (Illumina®). The samples were sequenced on the HiSeq 2500, and the resulting data was quantified using miRge2 (last change: 05/06/2018)^5^. MiRge2 used the following parameters: the miRBase database (miRBase v22) was used as the reference library and the bowtie binary was bowtie-1.1.125^6^. The output results were set in gff format. Although miRge2 outputs also the quantification and identification of isomirs, they were not utilized in this analysis. MiRNAs were linked to protein targets using the miRDB_hsa_v6.0 prediction result dataset, which provides a list of possible miRNA-mRNA inhibitory relationships, including a score that evaluates the probability of such inhibition.

For circRNA expression, we first extracted the putative spliced circRNA sequences from circBase in FASTA format and we added them into Salmon’s transcriptome reference, allowing us to obtain quantification from both linear and circular transcripts with standardized IDs in a single run. As circular RNAs have been hypothesized to be able to regulate miRNAs, after being able to connect miRNAs to the proteomics values, we were also able to connect the circRNA data to those miRNAs (and therefore, the target). To know which possible binding sites existed in circRNAs for miRNAs, we ran miRanda^7^ (version 3.3a, with the ‘-strict’ parameter for disabling G:U pairing with the 5' seed region) between the putative spliced circRNA sequences (circBase) and the mature miRNA sequences (miRbase). To evaluate the sponging effect power between a given circRNA and specific miRNA, we used the number of iterations predicted by miRanda.

# Bibliography

1 Selevsek, N. *et al.* in *Communications Biology* Vol. 3 1-15 (Nature Research, 2020).

2 Yen, H. C. S., Xu, Q., Chou, D. M., Zhao, Z. & Elledge, S. J. in *Science* Vol. 322 918-923 (Science, 2008).

3 Bolger, A. M., Lohse, M. & Usadel, B. in *Bioinformatics (Oxford, England)* Vol. 30 2114-2120 (Bioinformatics, 2014).

4 Patro, R., Duggal, G., Love, M. I., Irizarry, R. A. & Kingsford, C. in *Nature methods* Vol. 14 417-419 (Nat Methods, 2017).

5 Baras, A. S. *et al.* in *PLOS ONE* Vol. 10 (ed Wei Yan) e0143066 (Public Library of Science, 2015).

6 Langmead, B., Trapnell, C., Pop, M. & Salzberg, S. L. in *Genome Biology* Vol. 10 R25 (BioMed Central, 2009).

7 John, B. *et al.* in *PLoS Biology* Vol. 2 (Public Library of Science, 2004).
